# Ensemble Composition and Activity Levels of Insectivorous Bats in Response to Management Intensification in Coffee Agroforestry Systems

**DOI:** 10.1371/journal.pone.0016502

**Published:** 2011-01-26

**Authors:** Kimberly Williams-Guillén, Ivette Perfecto

**Affiliations:** University of Michigan, School of Natural Resources & Environment, Ann Arbor, Michigan, United States of America; University of Lancaster, United Kingdom

## Abstract

Shade coffee plantations have received attention for their role in biodiversity conservation. Bats are among the most diverse mammalian taxa in these systems; however, previous studies of bats in coffee plantations have focused on the largely herbivorous leaf-nosed bats (Phyllostomidae). In contrast, we have virtually no information on how ensembles of aerial insectivorous bats – nearly half the Neotropical bat species – change in response to habitat modification. To evaluate the effects of agroecosystem management on insectivorous bats, we studied their diversity and activity in southern Chiapas, Mexico, a landscape dominated by coffee agroforestry. We used acoustic monitoring and live captures to characterize the insectivorous bat ensemble in forest fragments and coffee plantations differing in the structural and taxonomic complexity of shade trees. We captured bats of 12 non-phyllostomid species; acoustic monitoring revealed the presence of at least 12 more species of aerial insectivores. Richness of forest bats was the same across all land-use types; in contrast, species richness of open-space bats increased in low shade, intensively managed coffee plantations. Conversely, only forest bats demonstrated significant differences in ensemble structure (as measured by similarity indices) across land-use types. Both overall activity and feeding activity of forest bats declined significantly with increasing management intensity, while the overall activity, but not feeding activity, of open-space bats increased. We conclude that diverse shade coffee plantations in our study area serve as valuable foraging and commuting habitat for aerial insectivorous bats, and several species also commute through or forage in low shade coffee monocultures.

## Introduction

The role of matrix habitats – the areas surrounding fragments of undisturbed habitat – in biodiversity conservation has received increasing attention. Inter-patch dispersal occurs through the matrix of surrounding anthropogenic habitat; however, this matrix can vary drastically in its quality as alternate or dispersal habitat, impacting dispersal rates and hence the long term population stability of forest-dwelling organisms [1], [2]. Understanding how different management regimes in matrix habitats affect the presence and diversity of wildlife in anthropogenic landscapes is therefore key to the conservation of biodiversity in the tropics. In the Neotropics, relationships between management intensity and biodiversity have received particular attention in coffee agroecosystems, due to this crop's economic importance, dominance at mid-elevation habitats where little undisturbed forest remains, and the vegetational complexity and associated diversity observed in traditional cultivation systems [3], [4]. Shade coffee plantations contain high diversity and abundance of arthropods, epiphytes, birds, and terrestrial vertebrates [4], [5], [6], [7].

Several recent studies have examined the diversity of bats in shade coffee [8], [9], the most species-rich mammalian order in tropical Central America [10]. Although the ability of many bats to enter areas without tree cover makes some species less vulnerable to fragmentation, several investigators have described the sensitivity of Neotropical bats to anthropogenic habitat change [8], [11], [12], [13], [14]. However, these studies have all focused on the largely herbivorous leaf-nosed bats (Family Phyllostomidae). This bias results from the relative ease with which phyllostomid bats are captured with mist nets: due to their low-intensity echolocation calls, leaf-nosed bats are less likely to detect and avoid nets [15]. Since mist nets are more readily available than other survey methods, they have provided the bulk of the data in studies of Neotropical bats – thus the responses to habitat change of approximately 50% of the region's bat species remain largely uninvestigated.

In Mesoamerica, the majority of non-phyllostomid bats are aerial insectivores: these bats use high-volume echolocation calls to locate and capture prey on the wing [16]. The majority of Neotropical aerial insectivores emit calls that can be recorded and identified with bat detectors and call visualization software [17]. Only recently have improvements in bat detector technology and the availability of reference calls allowed researchers to apply these techniques in the study of responses of insectivorous bats to habitat modification in the Neotropics. Working in a fragmented landscape in tropical Mexico, Estrada et al. [18] found that the activity levels of insectivorous bats were generally high in several agricultural and forested land-use types, but dropped dramatically in pastures; however, the authors did not differentiate between bat species. In the Yucatán Peninsula, MacSwiney et al. [19] recorded more activity in habitats with cenotes, with some species recorded exclusively at these water bodies; however, they recorded more bat activity in pasture versus forest. Jung and Kalko [20] recorded low activity levels of Panamanian bats at forest interior versus urbanized areas, although several species were limited to forests.

Differences in wing morphology and call structure affect how well bats can forage in space with dense vegetation (i.e., cluttered space; [16]). Estrada-Villegas et al. [21] investigated the responses of two functional groups of bats (open-space foragers versus forest foragers; the latter are adapted to foraging in background-clutter and high-clutter space) to differences in forest fragment size and isolation on islands of differing size and isolation in the Panama canal. For forest bats, they found differences in ensemble structure between treatments and reduced feeding activity on isolated islands; conversely, open-space foragers showed no differences in ensemble structure, and increased feeding activity on both small and isolated islands. In the context of terrestrial matrix habitat, agroecosystems with increased tree cover would have higher clutter.

In sum, the few studies of habitat use by Neotropical aerial insectivores suggest that responses to habitat change are idiosyncratic between species and functional groups; that anthropogenic habitat change may not necessarily have negative impacts on some of these bats; and that information on leaf-nosed bats therefore cannot substitute for detailed study of insectivore responses to land-use change. Additionally, none of the more detailed studies systematically investigated agricultural areas of varying management intensity, even though agriculture comprises most of the matrix in the tropics [22]. Since insectivorous bats limit insects in agricultural areas [23], [24], maintaining their populations in agroecosystems both supports biodiversity conservation and provides an important ecosystem service to farmers.

To our knowledge, this is the first detailed investigation of the diversity and activity of aerial insectivorous bats in coffee plantations. In this study, we explore whether the ensemble of aerial insectivorous bats is negatively impacted by reduced diversity and density of shade trees in coffee plantations. Following Estrada-Villegas et al. [21], we made the following predictions for two functional groups (forest versus open-space) of bats: (1) we expected forest bat species richness and activity levels to decline with increasing agricultural intensification; and (2) we expected open-space bat richness and activity levels to increase or show no response across the intensification gradient. Based on patterns observed for phyllostomid bats [25], we also expected to see the strongest responses in the most intensively managed coffee (i.e., plantations with monocultures of introduced shade trees). Because insects are more abundant in less intensively managed plantations, we also expected that feeding activity of both groups would be higher in less-intensive land-uses.

## Results

Over 44 nights we documented 24 species of non-phyllostomid bats belonging to five families (Table 1, Table S1). We captured 152 non-phyllostomid bats belonging to 12 species; only two individuals were open-space bats. A single vespertilionid species, *Myotis keaysi*, accounted for 58% of captures and was the most frequently captured insectivore in all land-use types (Table S1). Two other insectivorous species, *Pteronotus parnelli* and *Rhogeessa tumida*, comprised over 10% of total captures. Only one bat species, *Natalus stramineus*, was represented in captures but not in acoustic monitoring.

**Table 1:** Bat calls recorded per night (mean±SE) in forest fragments and shade coffee plantations in Chiapas, Mexico.

		Foraging	Call ID	Mean Passes per Night			
Family	Species	Habitat^a^	Source^b^	FF (N = 5)	LMC (N = 6)	MMC (N = 5)	HMC (N = 6)
Emballonuridae	*Balantiopteryx plicata*	UC	2, 7	–	–	2.20±2.20 (11)	0.17±0.17 (1)
Emballonuridae	*Diclidurus albus*	UC	7	–	2.50±2.31 (15)	1.00±0.77 (5)	0.17±0.17 (1)
Emballonuridae	*Peropteryx kappleri*	UC	3, 4, 7	–	–	DS	0.50±0.50 (3)
Emballonuridae	*Peropteryx macrotis*	UC	3, 5, 6, 7	6.00±6.00 (30)	5.83±3.90 (35)	13.20±7.33 (66)	16.00±7.95 (96)
Emballonuridae	*Saccopteryx bilineata*	BC	2, 3, 4, 5, 7	7.40±5.27 (37)	4.50±1.73 (27)	DS	11.50±9.99 (69)
Mormoopidae	*Mormoops megalophylla*	BC	2, 3, 4, 5, 6	–	–	0.20±0.20 (1)	0.33±0.33 (2)
Mormoopidae	*Pteronotus davyi*	BC	2, 3, 4, 5, 6	–	0.17±0.17 (1)	–	0.17±0.17 (1)
Mormoopidae	*Pteronotus parnelli*	HC	1, 2, 3, 4, 5, 6	10.80±7.18 (54)	4.83±2.44 (29)	6.80±5.56 (34)	0.17±0.17 (1)
Molossidae	*Cynomops mexicanus*	UC	3	0.20±0.0.20 (1)	0.67±0.49 (4)	1.80±0.86 (9)	4.17±1.74 (25)
Molossidae	*Eumops spp.* c	UC	3	1.60±0.75 (8)	1.33±0.21 (8)	3.20±1.36 (16)	6.50±2.59 (39)
Molossidae	*Molossus molossus*	UC	3, 4	2.44±0.89 (6)	0.33±0.33 (2)	2.00±1.14 (10)	1.67±1.17 (10)
Molossidae	*Molossus rufus*	UC	3, 4, 5	5.20±2.18 (26)	3.17±2.97 (19)	0.40±0.40 (2)	0.50±0.50 (3)
Molossidae	*Molossus sinaloae*	UC	3, 4, 5	0.20±0.20 (1)	–	0.40±0.40 (2)	2.00±2.00 (12)
Molossidae	*Nyctinomops laticaudatus*	UC	3, 5	0.20±0.20 (1)	–	0.40±0.24 (2)	–
Molossidae	*Promops centralis*	UC	5	DS	–	0.40±0.40 (2)	–
Vespertilionidae	*Eptesicus furinalis*	BC	1, 2, 3, 4, 5, 6	39.20±23.76 (196)	24.33±19.11 (146)	0.20±0.20 (1)	0.50±0.34 (3)
Vespertilionidae	*Lasiurus blossevillii*	BC	3	3.00±1.64 (15)	5.67±2.60 (34)	4.20±1.66 (21)	5.17±4.77 (31)
Vespertilionidae	*Lasiurus ega*	BC	3, 4, 5	0.80±0.58 (4)	1.33±0.88 (8)	0.40±0.24 (2)	0.83±0.31 (5)
Vespertilionidae	*Lasiurus intermedius*	BC	3, 4, 5, 6	2.40±2.40 (12)	0.17±0.17 (1)	–	2.17±2.17 (13)
Vespertilionidae	*Myotis elegans*	BC	1, 3, 4	5.00±3.16 (25)	2.50±1.73 (15)	–	–
Vespertilionidae	*Myotis keaysi*	BC	1, 2, 3, 4, 5, 6	123.00±30.05 (615)	136.33±30.51 (818)	101.60±49.34 (508)	53.00±12.65 (318)
Vespertilionidae	*Myotis nigricans*	BC	1, 8	29.40±27.17 (147)	1.83±0.95 (11)	8.60±4.02 (43)	1.83±1.64 (11)
Vespertilionidae	*Rhogeessa tumida*	BC	1, 2, 3, 4	46.80±14.90 (234)	115.50±60.05 (693)	84.40±24.55 (422)	11.50±3.71 (69)

Land-use types are forest fragments (FF), low-management coffee (LMC), medium-management coffee (MMC), and high-management coffee (HMC). Call frequency data are from wet season 2007 only. Numbers in parentheses indicate total number of detections during 172.4 hours passive Anabat monitoring, and 20.3 hours of active Pettersson monitoring; “DS” indicates species recorded during dry season during 214.7 hours of passive Pettersson monitoring.

a. Foraging habitat: UC, uncluttered (open) space; BC, background cluttered space; HC, highly cluttered space. Because only one species (*Pteronotus parnelli*) is classified as an highly-cluttered space forager, we combined BC and HC foragers into the forest bat group; classification from Schnitzler and Kalko [16] and Jung et al. [40].

b. Sources for call identifications: 1, Authors’ recordings from Chiapas, Mexico; 2, Authors’ recordings from other sites in Central America; 3, Miller [41]; 4 O’Farrell et al. [17]; 5, MacSwiney et al. [15]; 6, Rydell et al. [49]; 7, Jung et al. [40]; 8, Siemers et al. [50].

c. Probably *Eumops hansae* (captured on one occasion) and *Eumops underwoodi*.

During the dry season, we recorded 2,576 identifiable bat passes (2.2±3.9 SD passes per 10 minutes) belonging to 18 species from 4 families. During the wet season we recorded 5,196 identifiable passes (4,744 with the Anabat, 452 with the Pettersson); an additional five species were recorded during the wet season. We recorded an average of 5.0±3.6 SD identifiable passes per 10 minutes with the Anabat, and 4.4±3.0 SD passes per 10 minutes with the Pettersson; call rates recorded with passive and active monitoring were highly correlated (Table S2). Call rates also correlated with nightly capture rates (Table S2). Considering call rates from the wet season, acoustic monitoring data (Table 1) resemble the capture data (Table S1) in that *Myotis keaysi* was the most frequently recorded species (43.4% of passes), and *Rhogeessa tumida* the second-most recorded (27.3% of passes). However, several frequently recorded species were rarely or never captured. Twelve open-space bat species – all emballonurids or high-flying molossids – were documented only through acoustic monitoring.

**Figure 1:**
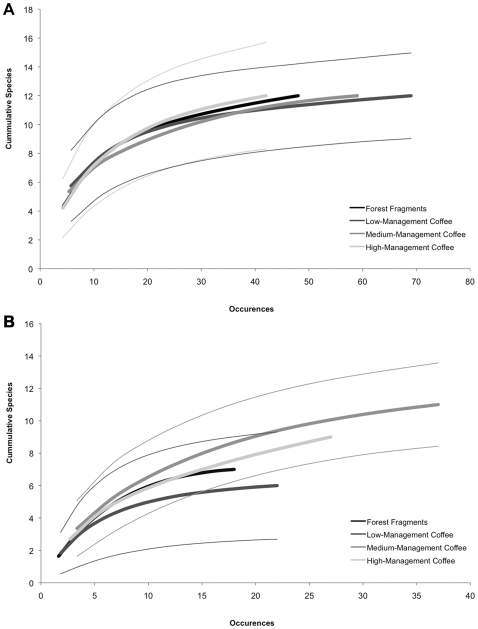
Occurrence-based species rarefaction curves of forest fragments and three coffee plantation management types. Species accumulation curves for forest (panel A) and open-space (panel B) bats. Thick lines indicate species accumulation curves for 1000 randomizations calculated using EstimateS. Thin lines indicate 95% confidence intervals for accumulation curves of corresponding color. Confidence intervals overlapped for both forest and open-space bats, suggesting no significant differences between land-use types in the species richness of these two groups of aerial insectivores; for the sake of clarity we present confidence intervals for only the upper and lower curves.

Few species were limited to just one or two land-use types (Table 1); in all cases these species were so infrequently encountered that presence cannot be interpreted as indicative of habitat preferences. Considering all data sources together, the landscape as a whole is estimated to contain 25 non-phyllostomid species, suggesting that we have adequately sampled aerial insectivores in the region (Table 2). Neither forest nor open-space bats showed significant differences in species richness between land-use types (Fig. 1), although more species of open-space bats were detected in intensively managed plantations (Table 2). The species composition (as assessed by Sorensen's index) of forest bats differed significantly between seasons (R = 0.204, *p* = 0.005) and land-use types (R = 0.112, *p* = 0.031), with significant differences between high-management versus low- and medium-management coffee. Although species composition of open-space bats differed between seasons (R = 0.186, *p* = 0.026) there were no significant differences between land-use types (R = 0.053, *p* = 0.174).

**Table 2:** Species richness of aerial insectivorous bats in forest fragments and coffee plantations in Chiapas, Mexico.

Measure	FF	LMC	MMC	HMC	All Sites
Total Species Observed					
Forest Bats	12	12	12	12	13
Open-Space Bats	7	6	11	9	11
Estimated Species Richness					
Forest Bats	13.2	12.8	13.1	13.3	13.4
Open-Space Bats	7.7	6.5	12.4	10.5	11.4
Inventory Completeness					
Forest Bats	90.9%	93.8%	91.6%	90.2%	97.0%
Open-Space Bats	90.9%	92.3%	88.7%	85.7%	96.5%

Observed and bootstrap estimated species richness calculated using all capture and call data, based on nightly presence/absence. Land-use types are forest fragments (FF), low-management coffee (LMC), medium-management coffee (MMC), high-management coffee (HMC), and all sites combined in southern Chiapas, Mexico.

**Figure 2:**
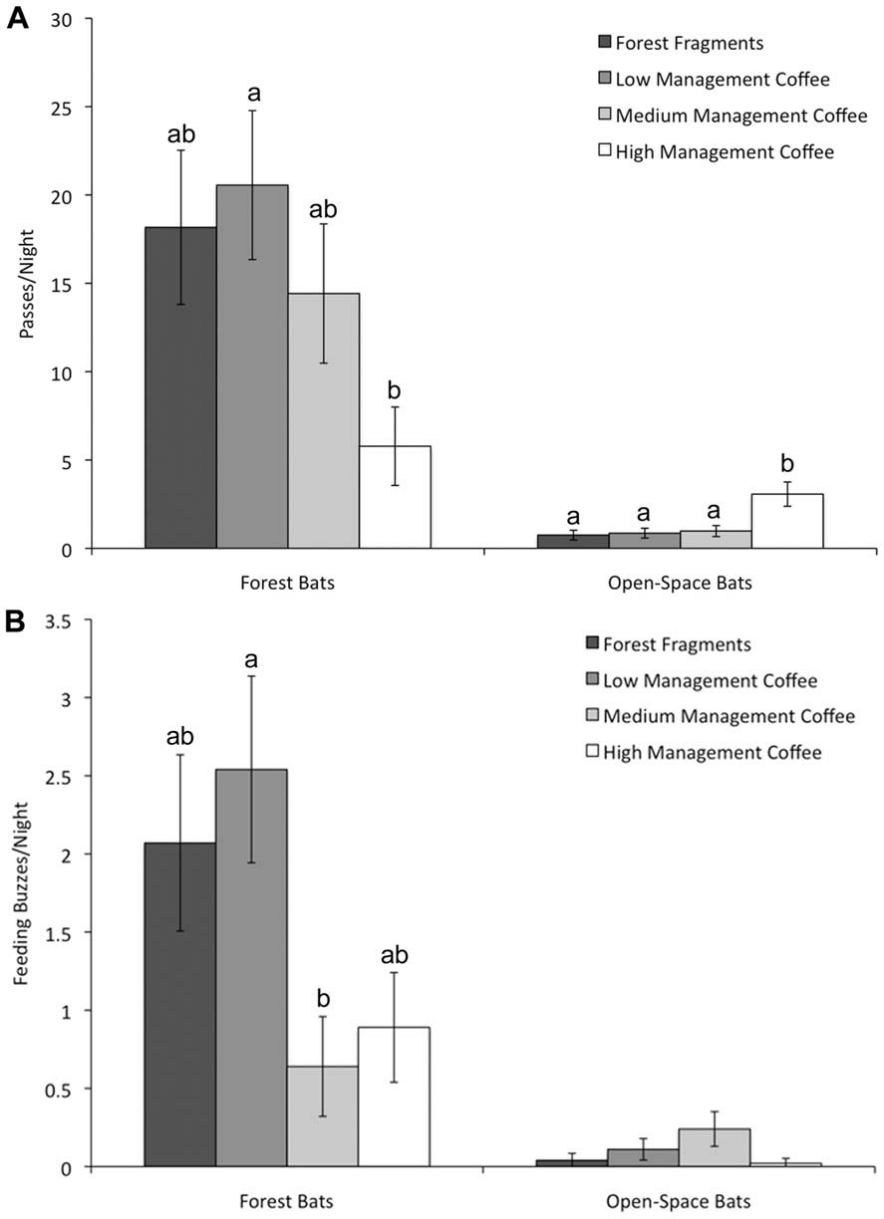
Relative activity of aerial insectivorous bats across land-use types. Mean±SE of passes per night (panel A) of forest and open-space bats; and feeding buzzes per night (panel B) of forest and open-space bats. Means are estimated marginal means (calculated using generalized linear models and incorporating significant covariates of cloud cover and elevation for pass rates of open-space bats). Means with different letters were significantly different (sequential Bonferroni, *p*<0.05).

Relative abundances (as measured by captures per mist-net hour) and activity levels showed much stronger differences between land-use types. We captured significantly more forest bats per night in the wet season than in the dry season (*D* = 42.652, df = 1, *p*<0.001); on average, we captured an average of 2.1±1.8 SD bats per night in the dry season, versus 4.8±4.6 SD bats per night in the wet season. Although more forest bats were captured per night in forest fragments and low-management coffee (Table S1), differences in captures across land-use types only approached significance (*D* = 6.780, df = 3, *p* = 0.079). Cloud cover also explained a significant portion of differences in capture numbers (*D* = 14.962, df = 1, *p*<0.001), with fewer captures on nights with intermediate cloud cover and more captures on nights with <10% or >80% cloud cover. Numbers of passes recorded per night of forest bats did differ significantly between land-use types (*D* = 11.385, df = 3, *p* = 0.010), with significantly more calls in low-management than high-management coffee (Fig. 2). We also found significant differences in passes per night for open-space bats (*D* = 13.906, df = 3, *p* = 0.003); however, open-space bats had an opposite pattern, with significantly more calls in high-management coffee versus all other land-use types (Fig. 2). For open-space bats, cloud cover (*D* = 13.255, df = 1, *p*<0.001) and elevation (*D* = 6.696, df = 1, *p* = 0.010) also explained a significant amount of variation in passes per night, with fewer calls at higher elevations or on nights with high cloud cover.

We observed significant differences across land-use types in the rates of feeding buzzes produced by forest (*D* = 11.506, df = 3, *p* = 0.009) but not open-space bats (*D* = 5.840, df = 3, *p* = 0.120); forest bats produced significantly more feeding buzzes in low-management versus medium-management coffee. The distribution of feeding buzzes across the gradient differs from those of acoustic counts (Fig. 2): for forest bats, medium and high-management coffee have similarly low feeding buzz rates. For open-space bats, although the highest numbers of passes were detected in high-management coffee, these plantations had the lowest foraging activity as measured by numbers of feeding buzzes.

## Discussion

We found that agricultural intensification in coffee plantations had considerable impacts on the relative activity of aerial insectivorous bats, and that bats' foraging adaptations explained the direction of response. Bats adapted to foraging in high-clutter and background-clutter environments (i.e., forest fragments and low-management coffee) showed significant changes species composition across the intensification gradient, and demonstrated significantly reduced overall activity and feeding activity – but not species richness – with increasing agricultural intensification. In contrast, bats adapted to foraging in low-clutter space (i.e., medium- and high-management coffee) demonstrated higher species richness and significantly higher pass rates in more intensively managed coffee plantations; however, open-space bats showed no differences in ensemble structure and feeding rates across the intensification gradient. As predicted, reductions in shade tree diversity and structural complexity were associated with negative impacts on forest bat activity. Our results accord with the few published studies on Neotropical aerial insectivores, particularly with regard to the higher sensitivity of forest bats to habitat change. Pass rates were similar to those recorded by Estrada et al. [18] in a fragmented landscape in Veracruz, Mexico; they also recorded high pass rates in anthropogenic habitat with tree cover. Estrada-Villegas et al. [21] found few differences between mainland and island areas in species richness, but found differences in species composition for forest bats. Similarly, they found relative abundance of open-space foragers was lower in forest interiors, and that the feeding activity of forest bats was reduced with fragmentation of habitat into islands. Jung et al. [20] found increased activity of several forest-adapted bats in forested versus unlit urban areas, while they observed the opposite for several open-space bat species. Our results are least similar to those of MacSwiney et al. [19], who recorded more passes of several forest bats in pastures versus forests.

One goal of this investigation was to determine the extent to which responses to land-use change demonstrated by aerial insectivores mirror those of the more easily captured leaf-nosed bats (Phyllostomidae). In the case of cluttered-space foragers (forest bats), responses are similar to those observed for the phyllostomids in this region, whose abundance and richness declines with agricultural intensification in coffee plantations [25], [26]. Leaf-nosed bats are also adapted to foraging in cluttered environments, with wing morphologies allowing highly maneuverable flight and echolocation calls with broadband, multi-harmonic structures adapted for detecting complex obstacles at short distances [16], and it therefore is not surprising that these two bat groups should respond similarly. However, netting of phyllostomids would be a poor substitute for acoustic monitoring of open-space aerial insectivores, whose response was opposite to that of the forest-adapted bats.

Taken at face value, our results would seem to suggest that open-space bats show few negative responses to agricultural intensification; however, the observed pattern of increased richness and activity in low-shade plantations could result in part from differences in detection probabilities between habitats that would affect the detection probabilities of both forest and open-space bats. First, forest fragments had few or no roads and trails; such anthropogenic flyways attract increased bat activity [27]. Although we always placed detectors in the best flyways available in forest fragments, since these were smaller and had more cluttered vegetation, the lack of features concentrating bat activity could mean that lower call rates in forests were an artifact rather than reflective of lower activity levels. Additionally, detection distances were probably higher in the low-shade plantations, since the lack of clutter would result in reduced call attenuation. Since trees are shorter in MMC and LMC, open-space bats foraging above the canopy could be foraging lower over the ground, and thus closer to the detectors, increasing detection probability. If detection probabilities do vary in this manner across the intensification gradient, the effects of management intensification on forest bat activity would be even more detrimental than our results suggest, while the differences pass rates of open-space bats could be an artifact of increased probability of detecting these bats flying above the open canopy in MMC and LMC.

Nevertheless, we believe that the increased activity of open-space bats in low-shade plantations stems not simply from a methodological bias, but also reflects real differences across the agricultural gradient. That these high-flying, less maneuverable bats should prefer foraging in open habitats is not surprising. MacSwiney et al. [19] observed higher levels of activity for most molossid species in pastures versus forests, and Jung and Kalko [20] recorded more molossid activity at urban versus forest sites. The higher activity of open-space foragers on more isolated islands in Panama [21] speaks to their ability to travel long distances with relative ease.

However, concluding that open-space bats reap unmitigated benefits from agricultural intensification would be unwarranted. Reduced levels of foraging activity in the most intensive plantations for both forest and open-space bats suggest that low shade coffee monocultures may serve more as commuting than foraging habitat. Additionally, many open-space bats use arboreal roosts [10], the availability of which declines in intensively managed agricultural areas [28]. Although many of these bats can fly long distances, loss of roosting areas at the landscape level should ultimately cause declines in bat populations regardless of the availability of food or preferred habitats.

Our results suggest several potential measures to conserve non-phyllostomid bats in our study area. First, as suggested by Struebig et al. [29] and Estrada-Villegas et al. [21], even small forest fragments have conservation value for insectivorous bats. Forest fragments probably provide better arboreal roosts than all but the most rustic shade coffee plantations. Many aerial insectivorous bats can readily use small and widely dispersed forest fragments, due to their ability to commute through a variety of matrix habitats (unlike the insectivorous phyllostomid bats, which avoid crossing open areas; [30]). Protecting these small fragments would enhance bat conservation at the landscape level. Secondly, given the greater sensitivity of forest bats to changes in tree cover, maintenance of a dense and diverse shade canopy in coffee plantations would enhance bat movement throughout the landscape and provide increased foraging opportunities for forest bats, many of which feed on the most damaging insect pests of coffee (KWG, unpublished data).

The vagility of many aerial insectivores suggests that landscape heterogeneity at spatial scales within the foraging ranges of bats may enhance both the abundance of aerial insectivores, and, by extension, the ecosystem services they provide. Landscape heterogeneity at multiple spatial scales is critical to maintaining biodiversity in agricultural landscapes [31]; unfortunately, the role of such heterogeneity on bats in farmlands has received little attention. Bats and other vertebrates play surprisingly important roles in suppressing herbivorous arthropods [32]. Understanding how best to manage farmed landscapes to encourage foraging by these animals could therefore enhance agricultural productivity.

Our results also have relevance to current debates regarding the role of agricultural systems in conservation [33]. From the point of view of a Neotropical insectivorous bat, landscapes dominated by “wildlife-friendly” farming (fine grained patches with high spatial continuity, relatively low agricultural intensification) may provide preferable foraging and roosting opportunities than a “land sparing” approach (coarse grained patches with high contrast between land-use types, agricultural intensification used to offset losses of productive land set aside for conservation; [33]). Multiple social, political, and ecological considerations influence which model best suits a region when planning the integration of agricultural areas into landscape-scale conservation plans [33]. The fragmented nature of most tropical landscapes, coupled with widespread poverty and food insecurity, means that land-sparing approaches could potentially *increase* biodiversity loss in tropical countries [22]. Given the importance of managed habitats for Neotropical insectivorous bats – and the importance of these bats for managed habitats – we see a critical need for further investigation of these long understudied animals in a variety of habitats across Mesoamerica.

## Methods

### Study Sites

This study was carried out in the Soconusco region of Chiapas, Mexico, a coffee growing region of ∼80,000 ha. The landscape is a mosaic of traditional coffee agroforestry, intensive coffee agriculture, and small fragments of tropical montane rainforest persisting in areas too steep for coffee cultivation. We worked from a field station at Finca Irlanda (15°10′ N, 92°20′ W, elevation ∼1000 m asl, annual rainfall ∼4,500 mm), a diverse shade coffee farm. The immediate study area is dominated by shade coffee cultivation, with only small remnant forest patches existing in this matrix; the only large block of forest in the area is the El Triunfo Biosphere Reserve, located approximately 50 km northwest of Finca Irlanda. To control for local effects, study sites were closely situated in a ∼25 km^2^ area representing a variety of land uses and management intensities (Fig. S1); distances between capture sites varied from 27 m (within small forest fragments) to 5 km (mean 2136 m±166 SE; Fig. 1). Because forest fragments were small and limited to areas too steep and inaccessible for coffee cultivation, it was impossible to find distant capture sites within fragments (the implications of this potential lack of spatial independence are discussed below in “Statistical Analyses”). The maximum distance from any coffee plantation capture site to the nearest forest fragment was <2 km (range 70–1946 m, mean 779 m±109 SE). This is a highly mountainous region and even within our relatively small study area, elevation ranged from 634 to 1268 m.

We recorded bats in forest fragments and in coffee plantations of differing diversity and density of shade trees; these plantations represented the greatest possible range of management intensities in the area. Based on measures of tree species richness, density, basal area, and shade cover, we classified sites as belonging to one of four land-use categories: forest fragments, low-management coffee, medium-management coffee, and high-management coffee; a detailed description of vegetation survey methods and characteristics in the study sites can be found in Williams-Guillén & Perfecto [25]. These three coffee systems correspond roughly to traditional polyculture, commercial polyculture, and shade monoculture in the classification of Moguel and Toledo [3]. Low–management coffee plantations had a structurally and taxonomically diverse canopy of shade trees of mostly native species, high-management plantations had a sparse, single-layer shade canopy consisting primarily of planted *Inga* trees, while medium-management plantations had a shade canopy of intermediate diversity and structural complexity.

We studied aerial insectivorous bats at the same sites where we sampled phyllostomid bats, which were captured in abundance with nets [25]; recording time was always similar to time spent mist-netting at each site (1800 h to 0400 hours in the dry season, and 1800 h to 0200 h in the wet season), with recording suspended during high winds or heavy rain. We recorded for an average of 8.8±1.6 SD hours per night. Sampling was conducted over 22 nights during November, December, and January 2006 (dry season) and over 22 nights during May, June, and August 2007 (wet season). A lack of land-use types in some areas, weather, and logistical difficulties prevented us from sampling all land-uses equally; we collected 11 nights of data in forest fragments, 12 in low-management coffee, 11 in medium-management coffee, and 10 in high-management coffee.

### Acoustic Monitoring of Echolocating Bats

We used acoustic monitoring equipment to record echolocation calls of bats. During the first field season (dry season 2006), we recorded time-expanded calls using a Pettersson D240x bat detector (Pettersson Elektronik AB, Uppsala, Sweden; frequency range 10–120 kHz, bandwidth 8 kHz±4 kHz -6 dB, sampling frequency 307 kHz, resolution 8 bits). Monitoring was passive during the dry season: the Pettersson detector was mounted on a pole 1.2 m high, the microphone angled upward, and placed near our netting sites but far enough away from nets to not record vocalizations of captured bats. At all sites, the detector was placed along a probable flyway (i.e., a road or trail) in an area with overhanging vegetation; however, the size and level of clutter along these paths, and the degree of overhanging vegetation, did vary between land-use types. The detector was connected directly to a digital MP3 recorder (iRiver iFP-800 digital audio recorder, iRiver Inc., Irvine, CA; bit rate 160 kbps, sampling frequency 44.1 kHz), set to automatically record when the detector was playing back a time-expanded call. We recorded 1.7 second time expanded (x10) calls using the auto setting, with the unit on high gain to maximize reception range and the trigger set to high to reduce the number of triggers caused by insect noise. During the dry season, we recorded for an average of 8.8±1.6 SD hours per night using this set up.

While this strategy allowed us to monitor echolocation while simultaneously capturing bats, the Pettersson performed poorly as a passive bat detector in this environment. Of 5,465 files recorded, over 50% had to be discarded, primarily because insect calls triggered recordings. While the detector was playing back time-expanded recordings of non-target organisms, it could not detect passing bats. We therefore believe that these recordings can be used to establish the presence of detected species; however, since the degree of insect interference probably differed between sites, and since time-expansion systems cannot record data continuously, these data are not used to investigate differences in activity levels between sites.

During the subsequent field season (wet season 2007), we used a combination of limited active monitoring with the Pettersson detector and passive monitoring with an Anabat II detector (Titley Electronics, Ballina, Australia; frequency range 10–200 kHz, bandwidth 20–2000 kHz, division ratio 16). Because we were also capturing bats concurrently, we limited active monitoring with the Pettersson detector was limited to one 10-minute recording session per hour [cf. 34] over a period of 6 hours. This approach combines the advantages of time expansion (high-detail recordings incorporating harmonic features, which can facilitate identification; [35]) and zero crossings detectors (smaller file size, time stamps, and real-time recording; [36]). The Anabat detector was positioned as described for the Pettersson passive monitoring; calls were recorded directly to a ZCAIM storage unit. Using the Anabat for passive monitoring, we recorded for an average of 7.4±1.3 SD hours per night. For active monitoring, the Pettersson detector was connected to the MP3 recorder as described above, but with the trigger set to manual. Once an hour, we walked along trails and roads in the vicinity of our trapping site, recording as many echolocation calls as possible. The ability to follow echolocating bats with the microphone greatly enhanced call quality and hence identifications, while moving between available microhabitats improved chances of detecting bat species not flying in the vicinity of the fixed location passive monitoring unit. Using this method, we recorded for an average of 51.3±11.4 SD minutes per night. We combined data from the two sources for analyses.

Files recorded with the Pettersson detector were analyzed with SonoBat v. 2.6 (DNDesign, Arcata, CA). Files recorded with the Anabat were analyzed with AnalookW v. 3.5a (Titley Scientific, Ballina, Australia). For both recording methods, we defined a pass as a sequence of at least two successive echolocation pulses [37]. We considered each file to have only one pass of a given species, even if long gaps between pulse series suggested multiple flights past the microphone. However, due to the diversity of bats at our study sites, it was common to record multiple species on the same file; in these cases, a pass was counted once for each of the species represented. Pass rates are used to contrast relative activity (passes per night) between land-use types. Call sequences were then inspected for feeding buzzes (indicating prey capture) by examining call sequences in real time view, and, for Pettersson files, with the audio playback feature in SonoBat; the rate of feeding buzzes is used as an estimator of relative feeding activity. We emphasize that pass rates, while probably correlated with raw abundance, may not accurately reflect abundance [38]. A recording of one pass of a given species definitely represents one individual; ten recordings could represent ten individuals, one individual passing the detector ten times, or some intermediate number of bats. Raw pass rates should therefore be considered reflective only of relative activity (rather than abundance) in a given land-use type.

Passes were assigned species identifications through visual inspection of pulse sequences [39]. Identifications were made by inspecting individual pulses and pulse sequences for key features, primarily pulse shape and bandwidth, frequency of maximum energy (characteristic frequency for Anabat calls), terminal frequency, minimum frequency, and pulse duration. For calls analyzed in Analook, the sequence of pulses in question was selected (excluding poor quality or fragmented pulses), and the analysis function was used to automatically determine characteristic frequency, minimum and maximum frequencies, pulse duration, etc. For pulse sequences analyzed with SonoBat, we selected the highest-quality pulse in the sequence (i.e., good signal-to-noise ratio, broad bandwidth, no apparent missing frequency components, and with harmonics when possible). Using the analysis tool in SonoBat, we manually placed the cursors on the time-frequency sonogram of the selected call to determine minimum, maximum, maximum energy frequencies, and pulse duration. We were also able to inspect call harmonics to further support identification. SonoBat adjusts the FFT parameters to optimize the display and uses different parameters depending on display options; quantitative measurements were made using the standard view display, which uses 1024 frequency bins with a 256 point Hanning window and an 8 point time interval (18.1 microseconds with analysis performed at 44.10 kHz for 10x time expansion; Szewczak, personal communication). A minimum of two pulses was required to identify bats with easily recognizable call structures (e.g., *Pteronotus parnelli*); we usually required ≥5 complete pulses to identify vespertilionids. We used published information on call parameters (see Table 1 for sources) and a call library we developed from hand released and zip-lined bats. Passes to which we could not confidently assign a species (faint or fragmentary calls, or calls lacking structure easily characterized to species, e.g. *Natalus stramineus*) were excluded from further analyses (16% of 486 passes recorded with active monitoring and 13% of 5,466 passes recorded with the Anabat). Given that bat calls can be highly variable within species, and that the calls of many Neotropical species have not been well documented – particularly for molossids – it is likely that the true number of species was underestimated. Identified calls were then assigned to the open-space versus forest functional groups based on the classifications described by Schnitzler and Kalko [16] and Jung et al. [40].

The calls of the mormoopid species known from our study area are highly distinctive, and presented little problem in species assignment. Similarly, calls of most emballonurids were assigned to species with relative ease. Two species provided some difficulty in classification. The call parameters of *Diclidurus albus* overlap with those of *Eumops*. Calls were assigned to *D. albus* only if the duration of complete (not fragmentary) pulses was less than the known range for *Eumops*, or if call sequences showed a stepped pattern [40]. While *Peropteryx macrotis* was the most frequently recorded emballonurid, *Balantiopteryx plicata* was recorded on only a handful of occasions. These two species have similar echolocation calls, but those of *B. plicata* had a characteristic frequency of 42 kHz or greater [40].

Characterizing some molossid calls was difficult due to the lack of reference calls; we therefore grouped together all probable *Eumops* calls [20], [21], even though multiple species are almost certainly included in this one category (most likely at least *Eumops underwoodi* and *E. hansae*; the latter was captured on one occasion). It is possible that calls identified as *Nyctinomops laticaudata* include multiple species of *Nyctinomops*; however, all calls conformed to published parameters for *N. laticaudata*. Calls from bats of the genus *Molossus* could frequently be assigned to a species due to the stepped patterns and non-overlapping peak frequencies. A number of highly unusual (Fig. S2) molossid calls were recorded, primarily in forest fragments; these resemble calls identified by MacSwiney et al. [15] as potentially belonging to *Promops centralis*. Because the presence of this bat species was confirmed via a captured individual, we classified these calls as *P. centralis.* Finally, we did not assign species-level identifications to several sequences belonging to molossids (5% of 324 molossid passes recorded) because they did not unambiguously match call parameters described in the literature; these calls are included in calculations of relative abundance of open-space foraging species, but not in estimates of species richness and similarity.

Differentiating between vestpertilionid species was particularly difficult; we relied primarily on the minimum frequency and pulse duration [41] to assign species identifications. *Lasiurus spp.* were identified based on their call ranges (lower than *Eptesicus furinalis* for *L. ega* and *L. intermedius*, higher for *L. blossivillii*) and undulating minimum frequencies within the same call sequence. We differentiated between *L. ega* and *L. intermedius* based on the minimum frequency (<30 kHz for *L. intermedius*, >30 kHz for *L. ega*). Identification of vespertilionid calls with minimum frequencies between 48–58 kHz proved most challenging, due to overlap in the call parameters of *Rhogeessa tumida*, *Myotis nigricans*, and *Myotis keaysi*. Of these three species, *R. tumida* and *M. keaysi* were frequently captured, and we therefore assigned vespertilionid calls with a minimum frequency of ≤53 to *R. tumida* (the upper range of its peak frequency) and >53 to *Myotis keaysi* (the lower range of its peak frequency), unless the pulse duration exceeded the reported limits for these species (i.e., only calls with pulse durations of ≥6.5 ms were assigned to *M. nigricans*). This may result in underestimates of the relative activity of *M. nigricans.* It should also be noted that repeated recordings of *Myotis keaysi* at our study sites from hand released, ziplined, and free-flying individuals marked with light tags all suggested that in our study area *M. keaysi* calls typically had a peak frequency of 55–56 kHz, somewhat lower than frequencies reported for this species at other sites [15], [17], [41].

### Bat Captures

Concurrent with acoustic monitoring, we captured bats. Although acoustic monitoring is more effective for characterizing ensembles of aerial insectivores because many insectivores can readily detect and avoid nets or fly well above ground level [15], captures provide information on species that cannot be recorded, and allowed us to asses the degree of concordance between capture and acoustic monitoring data. We captured bats with mist nets and identified bats as described in Williams-Guillén & Perfecto [25]. Bats were marked on the wing with a silver sharpie to prevent data replication due to recaptures on the same night; however, these bats were not marked with a permanent method, and there is a possibility that some individuals were recaptured at other sites. We also used two 1.8 m×1.8 m harp traps, which are more effective than mist nets in capturing echolocating insectivores [42]. To account for variable sampling effort, we standardized captures by total m^2^ hours (a 12-m long and 2.6-m high mist net open for one hour would represent 31.2 m^2^ hours, a harp trap 3.24 m^2^ hours); capture rates serve as an estimator of relative abundances between land-use types and seasons [43]. Because capture rates with mist nets and harp traps were significantly correlated for forest bats (Table S2) we combined data from these two capture devices; captures of open-space bats were so infrequent that they could not be analyzed.

### Statistical Analyses

For all analyses, we followed Estrada-Villegas et al. [21] in using separate analyses for open-space versus forest bats (see Table 1 for species in each functional group). The limited study sites available in the forest fragments resulted in closely situated capture and monitoring sites, which could result in spatial non-independence of samples. However, Mantel tests contrasting pair-wise geographic distance and Sorensen dissimilarity demonstrate no relationship between proximity and similarity for forest (R = 0.030, *p* = 0.273) or open-space bats (R = 0.041, *p* = 0.183). We therefore treat each night as an independent sample for statistical analyses.

We used non-parametric Spearman rank correlations to explore relationships between relative activity levels and abundances measured with different capture and acoustic monitoring methods (Table S1). Data from captures and acoustic monitoring were pooled for analyses of species richness and similarity. Because calls do not represent individual bats (the same individual could be sampled multiple times, and similar numbers of individuals sometimes can produce highly divergent pass numbers; [38]), we use presence/absence data for diversity analyses. To compare species richness between land-use types, we used EstimateS version 8.0 [44] to generate species accumulation curves and 95% confidence intervals from 1000 randomizations. Curves were scaled to occurrences. We considered the bootstrap species richness estimator most appropriate for our data set (incidence based, no reliance on uniques or duplicates, and suitable for small sample sizes; [45]). Inventory completeness was calculated as the percentage of estimated species actually observed [46]. To test for significant differences in species composition between seasons and land-use types, we used a two-way Analysis of Similitude (ANOSIM) test, a non-parametric permutation test analogous to ANOVA using similarity indices [47]. ANOSIM tests were performed in PRIMER v.6 (PRIMER-E, Lutton, UK) using the Sorensen similarity index; nights with no bats captured or detected (1 night for forest bats, 6 nights for open-space bats) were excluded from similarity calculations.

To test for significant differences between land-use types and season (in the case of captures) in relative activity or abundances of bats, we used generalized linear models; we modeled data according to the distribution providing the best fit (Poisson corrected for overdispersion in all cases). Raw pass counts or capture numbers were used as response variables, with the log of total effort (total m^2^ hours for captures, total recording time for acoustic monitoring) used as the offset variable to account for different sampling efforts between nights [48]. During initial model testing we included environmental (temperature, relative humidity, wind speed, percent cloud cover) and landscape variables (elevation, distance to nearest forest fragment) as covariates in the analyses, successively eliminating the least significant covariates until only those with significant explanatory power remained. In all cases, exclusion of these non-significant covariates improved model fit as assessed with the corrected Akaike Information Criterion. We constructed a two-way model for capture data (season, land-use, and interaction) and a one-way model for acoustic monitoring data (land-use, using only wet season rates).

## Supporting Information

Figure S1 **Map of study region.** Locations of coffee plantations and forest fragments where surveys were conducted (shading indicates management intensity; lighter areas have less shade cover) and locations where bats were captured and calls recorded in each season.
(TIF)

Figure S2 **Representative call pulses of bats identified through acoustic monitoring.** Sonograms and oscillograms of representative call pulses of bats identified in this study; (a) Emballonuridae, (b) Mormoopidae; (c) Molossidae; (d) Vespertilionidae. Pulse intervals have been compressed.
(TIF)

Table S1 Captures (mean±SE) of aerial insectivorous bats per 1000 m^2^ hours of capture effort in forest fragments (FF), low-management intensity (LMC), medium-management intensity (MMC), and high-management intensity (HMC) shade coffee plantations in the Soconusco region of Chiapas, Mexico. Numbers in parentheses following means indicate total number of captures from 2104.6 12-m by 2.6-m mist-net hours and 683.0 1.8-m by 1.8-m harp trap hours.
(DOC)

Table S2 Spearman rank correlations between capture and acoustic monitoring variables. Relationships significant at the ≤0.1 level are indicated with bold text.
(DOC)
