# Supplementary figures and images for: Ensemble Composition and Activity Levels of Insectivorous Bats in Response to Management Intensification in Coffee Agroforestry Systems
Source: PLoS One. 2011 Jan 26;6(1):e16502. doi: 10.1371/journal.pone.0016502 (PMC3027674; doi:10.1371/journal.pone.0016502)

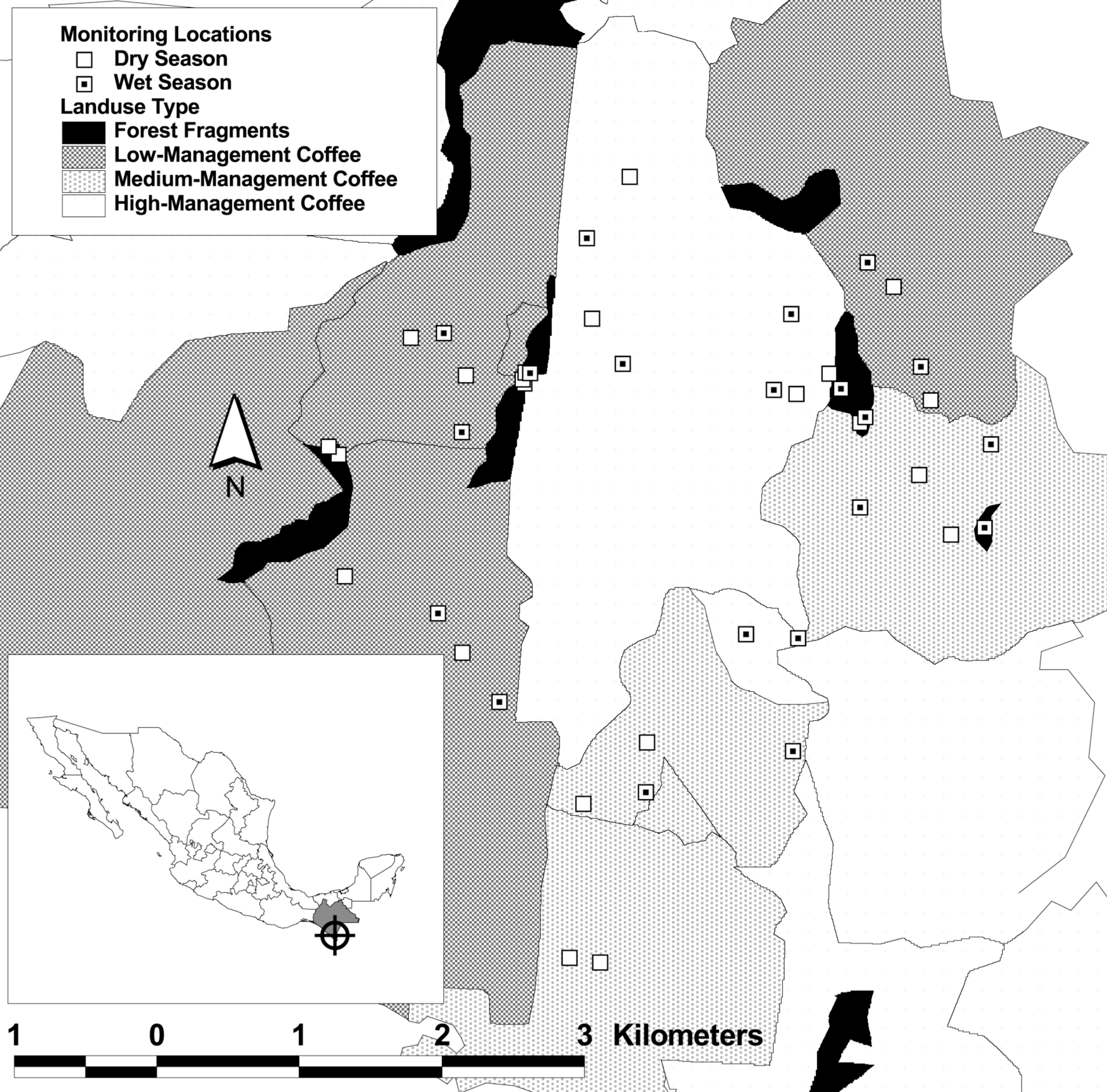

Supplement: Figure S1 — Map of study region. Locations of coffee plantations and forest fragments where surveys were conducted (shading indicates management intensity; lighter areas have less shade cover) and locations where bats were captured and calls recorded in each season. (TIF) [file pone.0016502.s001.tif]

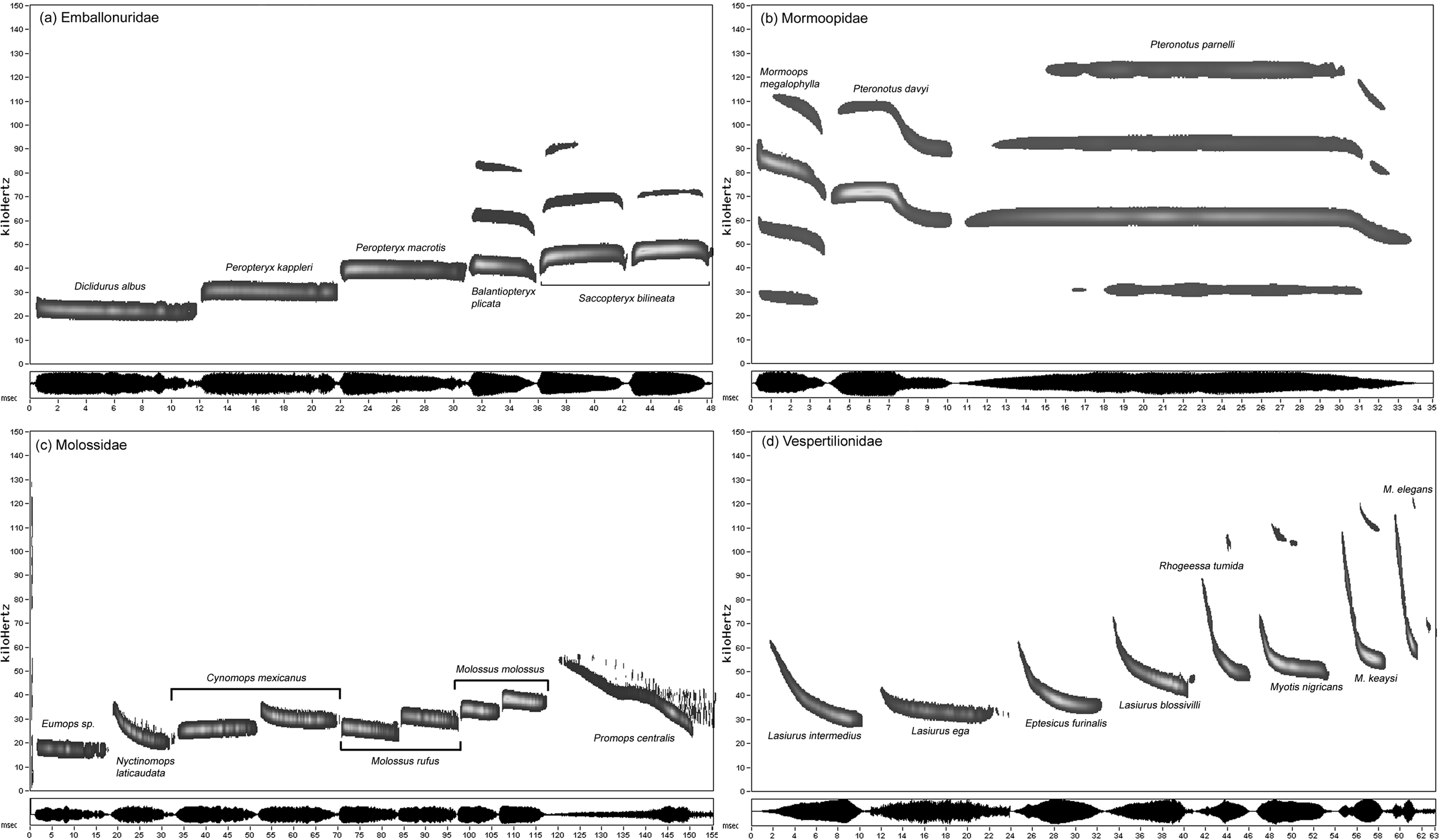

Supplement: Figure S2 — Representative call pulses of bats identified through acoustic monitoring. Sonograms and oscillograms of representative call pulses of bats identified in this study; (a) Emballonuridae, (b) Mormoopidae; (c) Molossidae; (d) Vespertilionidae. Pulse intervals have been compressed. (TIF) [file pone.0016502.s002.tif]
