# Supplementary material for: Ensemble Composition and Activity Levels of Insectivorous Bats in Response to Management Intensification in Coffee Agroforestry Systems
Source: PLoS One. 2011 Jan 26;6(1):e16502. doi: 10.1371/journal.pone.0016502 (PMC3027674; doi:10.1371/journal.pone.0016502)
Supplement: Table S1 — Captures (mean±SE) of aerial insectivorous bats per 1000 m2 hours of capture effort in forest fragments (FF), low-management intensity (LMC), medium-management intensity (MMC), and high-management intensity (HMC) shade coffee plantations in the Soconusco region of Chiapas, Mexico. Numbers in parentheses following means indicate total number of captures from 2104.6 12-m by 2.6-m mist-net hours and 683.0 1.8-m by 1.8-m harp trap hours. (DOC) [file pone.0016502.s003.doc]

**Table S1.** Captures (mean±SE) of aerial insectivorous bats per 1000 m^2^ hours of capture effort in forest fragments (FF), low-management intensity (LMC), medium-management intensity (MMC), and high-management intensity (HMC) shade coffee plantations in the Soconusco region of Chiapas, Mexico. Numbers in parentheses following means indicate total number of captures from 2104.6 12-m by 2.6-m mist-net hours and 683.0 1.8-m by 1.8-m harp trap hours.

|  |  |  | Captures/1000 m^2^-hours | | | |
| --- | --- | --- | --- | --- | --- | --- |
|  | Foraging  Habitat* |  | FF  (11 nights, 16,948 m^2^ hours) | LMC  (12 nights, 19,651 m^2^ hours) | MMC  (11 nights, 17,085 m^2^ hours) | HMC  (10 nights, 14,652 m^2^ hours) |
| Mormoopidae |  |  |  |  |  |  |
| *Mormoops megalophylla* | BC |  | 0.04±0.04 (1) | -- | -- | -- |
| *Pteronotus davyi* | BC |  | 0.04±0.04 (1) | 0.05±0.05 (1) | -- | -- |
| *Pteronotus parnelli* | HC |  | 0.99±0.95 (10) | 0.42±0.18 (6) | 0.16±0.08 (3) | 0.09±0.09 (2) |
| Natalidae |  |  |  |  |  |  |
| *Natalus stramineus* | BC |  | -- | 0.03±0.03 (1) | -- | -- |
| Molossidae |  |  |  |  |  |  |
| *Eumops underwoodi* | UC |  | -- | -- | 0.06±0.06 (1) | -- |
| *Promops centralis* | UC |  | -- | -- | 0.04±0.04 (1) | -- |
| Vespertilionidae |  |  |  |  |  |  |
| *Eptesicus furinalis* | BC |  | 0.13±0.13 (3) | 0.11±0.11 (2) |  |  |
| *Lasiurus blossevillii* | BC |  | 0.10±0.10 (1) | -- | 0.03±0.03 (1) | -- |
| *Myotis elegans* | BC |  | 0.09±0.06 (2) | 0.46±0.22 (3) | 0.14±0.11 (2) | 0.26±0.14 (3) |
| *Myotis keaysi* | BC |  | 2.09±0.86 (25) | 2.14±0.99 (31) | 1.40±0.63 (18) | 1.05±0.60 (12) |
| *Myotis nigricans* | BC |  | -- | 0.02±0.02 (1) | -- | -- |
| *Rhogeessa tumida* | BC |  | 0.11±0.11 (1) | 0.86±0.42 (11) | 0.69±0.29 (7) | 0.16±0.12 (2) |

* Foraging habitat: UC, uncluttered (open) space; BC, background cluttered space; HC, highly cluttered space; classification from Schnitzler and Kalko and Jung et al. .
