# Supplementary material for: Ensemble Composition and Activity Levels of Insectivorous Bats in Response to Management Intensification in Coffee Agroforestry Systems
Source: PLoS One. 2011 Jan 26;6(1):e16502. doi: 10.1371/journal.pone.0016502 (PMC3027674; doi:10.1371/journal.pone.0016502)
Supplement: Table S2 — Spearman rank correlations between capture and acoustic monitoring variables. Relationships significant at the ≤0.1 level are indicated with bold text. (DOC) [file pone.0016502.s004.doc]

**Table S2.** Spearman rank correlations between capture and acoustic monitoring variables. Relationships significant at the 0.1 level are indicated with bold text.

| **Contrast Type** | **Variable 1** | **Variable 2** | **N** | **r_s_** | ***p*** |
| --- | --- | --- | --- | --- | --- |
| Between capture methods (forest bats only) | Mist net capture rate | Harp trap capture rate | 44 | 0.334 | **0.028** |
| Between acoustic monitoring methods (wet season only) | Calls per night recorded with Anabat (forest bats) | Calls per night recorded with Pettersson (forest bats) | 22 | 0.681 | **<0.001** |
|  | Calls per night recorded with Anabat (open-space bats) | Calls per night recorded with Pettersson (open-space bats) | 22 | 0.395 | **0.069** |
|  | Species per night recorded with Anabat (forest bats) | Species per night recorded with Pettersson (forest bats) | 22 | -0.260 | 0.243 |
|  | Species per night recorded with Anabat (open-space bats) | Species per night recorded with Pettersson (open-space bats) | 22 | 0.501 | **0.018** |
| Between captures and acoustic monitoring (forest bats only) | Calls recorded per night (forest bats) | Bats captured per night (forest bats) | 44 | 0.427 | **0.004** |
|  | Species recorded per night (forest bats) | Species captured per night (forest bats) | 44 | 0.296 | **0.051** |
